# Electrospun, Oriented, Ferromagnetic Ni_*1-x*_Fe_*x*_ Nanofibers

**DOI:** 10.3389/fchem.2020.00047

**Published:** 2020-02-13

**Authors:** Vaibhav S. Bhugra, Fraser R. Hughson, Grant V. M. Williams, Shen V. Chong, Thomas Nann

**Affiliations:** ^1^School of Chemical and Physical Sciences, Victoria University of Wellington, Wellington, New Zealand; ^2^Robinson Research Institute, Victoria University of Wellington, Lower Hutt, New Zealand; ^3^School of Mathematical and Physical Sciences, The University of Newcastle, Newcastle, NSW, Australia

**Keywords:** magnetic properties, electrospinning, nanofibers, ferromagnetic, polyvinylpyrrolidone (PVP), nanomaterials

## Abstract

Electrospinning has been used to fabricate ferromagnetic Ni_0._47Fe_0.53_ nanofiber mats that were composed of individual, orientated Ni_0.47_Fe_0.53_ nanofibers. The key steps were processing a polyvinylpyrrolidone nanofiber template containing ferric nitrate and nickel acetate metal precursors in Ar at 300°C and then 95% Ar: 5% H_2_ at 600°C. The Ni_0.47_Fe_0.53_ fibers were nanostructured and contained Ni_0.47_Fe_0.53_ nanocrystals with average diameters of ~14 nm. The Ni_0.47_Fe_0.53_ ferromagnetic mats had a high saturation magnetic moment per formula unit that was comparable to those reported in other studies of nanostructured Ni_1-*x*_Fe_*x*_. There is a small spin-disordered fraction that is typically seen in nanoscale ferromagnets and is likely to be caused by the surface of the nanofibers. There was an additional magnetic contribution that could possibly stem from a small Fe_1-*z*_Ni_*z*_O phase fraction surrounding the fibers. The coercivity was found to be enhanced when compared with the bulk material.

## Introduction

There is extensive ongoing research into nanoscale magnetically ordered materials sparked by their potential applications in medicine (Pankhurst et al., 2003; Kim et al., 2007), magnetic sensors (Chen et al., 2011; Kennedy et al., 2014), RF components (Chinnasamy et al., 2015), magnetic fluids (Chen et al., 2011), and magnetic memory (Moser et al., 2002). Current research is driven by the fact that the properties of nanoscale ferromagnetic materials can be different from those seen in the bulk (Hendriksen et al., 1993; Batlle and Labarta, 2002; Goya et al., 2003; Upadhyay et al., 2016; Williams et al., 2019). For example, they have been reported to show enhanced coercivities that can be useful for magnetic memory storage devices (Barakat et al., 2009; Dong et al., 2014). The appearance of uncompensated moments or spin-disorder in the shell can lead to an exchange bias that can be used in magnetic random-access memory (RAM) (Katti, 2002; Nogués et al., 2005; Wu et al., 2013). It is also possible that the magnetocrystalline anisotropy can change particularly due to a large shell contribution that will affect the magnetic properties that include the saturation field (Batlle and Labarta, 2002; Goya et al., 2003; Demortière et al., 2011). They can even have an enhanced magnetostriction (Balaji et al., 2012), which is advantageous for composite multiferroics for applications that include magnetic sensors (Chong and Williams, 2019). If all three dimensions of the nanoscale materials are small enough, they can display superparamagnetism above a blocking temperature, *T*_*B*_, where the thermal energy is greater than the magnetocrystalline anisotropy energy (Cullity and Graham, 2009). The hysteresis is negligible above *T*_*B*_ (Cullity and Graham, 2009), which is advantageous for applications that include magnetic sensing where the appearance of hysteresis affects the repeatability and minimum detectible field.

Most research has focused on nanoscale materials where all three dimensions are small (Pankhurst et al., 2003; Chen et al., 2011; Kennedy et al., 2014). However, one-dimensional ferromagnetic materials in the shape of nanofibers are also particularly interesting. Their length and narrow diameters make them ideal for magnetic flux guiding applications on the nanoscale. It is also possible that they can have nanodomains extending across the diameter of the fiber and hence they could be used for nanoscale domain wall magnetic memory (Cisternas et al., 2017). Metallic and ferromagnetic nanofibers that display a degree of electronic spin polarization could also potentially be used in spin-tunneling junctions (Katti, 2002; Parkin et al., 2004; Williams et al., 2018) for magnetic RAM applications where their small size could lead to high density storage as well as lower power when compared with multilayer thin films. They could also be used for spin-tunneling nano-magnetic sensors where the long lengths in relation to the diameter can ensure flux guiding along the length of the fiber and hence magnetic sensing directionality provided that the magnetic permeability is high enough.

Electrospinning (Li et al., 2003; Graeser et al., 2007; Wu et al., 2007) and electrodeposition using an aluminum oxide or other template (Salem et al., 2012; Zhang et al., 2013; Meneses et al., 2018; Frolov et al., 2019) are two common methods to make ferromagnetic metallic nanofibers of materials that include Ni (Wu et al., 2007; Meneses et al., 2018), Fe (Graeser et al., 2007; Wu et al., 2007), Co (Graeser et al., 2007; Wu et al., 2007), and Ni_1-*x*_Fe_*x*_ (Salem et al., 2012; Zhang et al., 2013; Frolov et al., 2019). Electrospinning has the advantage, that it is, a relatively simpler preparation method and it is possible to fabricate larger nanofibre mats. The electrospinning process starts with preparing a polymer solution of the metal precursors, a polymer, and solvent. A high voltage is applied between the polymer solution and the collector to ensure that the electric field is large enough to result in a charged jet of the polymer solution that rapidly thins due to solvent evaporation and leads to solid fibers being deposited on the collector (Graeser et al., 2007). Continual movement of the collector leads to orientated nanofibers. Fe and Ni nanofibers have been made by electrodeposition, but we are not aware of any reports of Ni_1-*x*_Fe_*x*_ nanofibers being made by electrospinning. Ni_1-*x*_Fe_*x*_ is a well-known bimetallic material with a very large magnetic permeability and it can have a low magnetocrystalline anisotropy for *x* ~ 0.25 (Bozorth and Walker, 1953; Cullity and Graham, 2009). It also has a degree of electronic spin polarization (Žutić et al., 2004) that can lead to spin-dependent tunneling or spin-dependent scattering (Daughton et al., 1994; Inoue and Maekawa, 1996; Prakash et al., 2014), which is useful for magnetic sensors (Daughton et al., 1994). Electrospun Ni_1-*x*_Fe_x_ nanofibers that have a degree of orientation have a number of potential applications, that include very nanoflux guides that can be used, for example, for thin and compact wireless power transfer. By coating the nanofibers with a piezoelectric polymer (e.g., PVDF) it would be possible to create a magneto-electric composite as already shown for Fe_3_O_4_ nanoparticle/PVDF nanocomposites (Chong and Williams, 2019) that can find application in electrically tunable miniature RF filters and antennas (Petrov et al., 2008; Guo-Min et al., 2009).

In this paper we report the successful synthesis of Ni_1-*x*_Fe_*x*_ mats containing orientated submicron fibers with *x* = 0.53 by the electrospinning process followed by heat treatment and reduction in a 95% Ar:5% H_2_ atmosphere. This value of *x* was chosen because it is close to where the saturation magnetic moment is the highest while still maintaining the FCC crystal structure seen for *x* < ~ 0.6 (Li et al., 1997). We were able to show that our fibers were nanocrystalline and ferromagnetic and with a saturation magnetic moment that is comparable to that seen in other nanostructured Ni_1-*x*_Fe_*x*_ compounds with similar *x* values. We also showed that there is evidence for spin-disorder regions that may arise from the surface regions of the Ni_1-*x*_Fe_*x*_ fibers.

## Experimental Details

### Materials

All the chemicals used in the reaction were of analytical grade. The polyvinylpyrrolidone (PVP, M_w_ = 1,300,000—polymer for electrospinning), iron(III) nitrate nonahydrate (Fe(NO_3_)_3_.9H_2_O), >98%—precursor for Fe and nickel(II) acetate tetrahydrate (C_4_H_14_NiO_8_.4H_2_O), 98%—precursor for Ni were obtained from Sigma-Aldrich. Methanol was obtained from Chem Supply.

### Experimental Work

**Figure 1:**
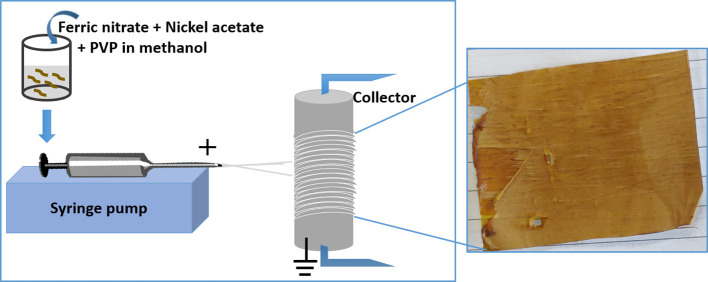
Electrospinning setup for making PVP/Fe nitrate/Ni acetate nanofibers.

In a typical procedure, 2 g (5 mmol) of Fe nitrate and 1.5 g (6 mmol) of Ni acetate were mixed in 10 ml of methanol and 0.5 g polyvinylpyrrolidone (PVP). The whole mixture was stirred overnight at 300 rpm and loaded into a 5 mL plastic syringe fitted with a 21-gauge stainless steel needle. The needle was connected to a high-voltage supply (Gamma High Voltage Research, Ormand Beach, FL). A rotating drum was chosen as a collector, which acted as a counter electrode to collect highly charged fibers and to create aligned fibers. The rotating drum was wrapped with aluminum foil prior to fiber collection, which is advantageous for post electrospinning treatment of the fibers and it also avoids mechanically induced damage. The syringe was attached to a syringe pump (Harvard Apparatus) as shown in Figure 1 and the solution was pumped out at a rate of 0.65 ml/h. The voltage for electrospinning was set to 17.5 kV and the distance between the electrodes was fixed to 10 cm. The sample was transferred into a tube furnace after electrospinning and heated in an argon atmosphere at a rate of 10°C/min and then held for 1.5 h at 300°C. This was then followed by increasing the temperature of the furnace at a rate of 10°C/min to 600°C in a 95% Ar:5% H_2_ atmosphere and holding for 3 h. The furnace was then switched off and allowed to cool down to room temperature. The resultant sample was black and brittle.

### Characterization

X-Ray diffraction (XRD) was performed on a Panalytical X-Ray diffractometer using Cu-K_α_ radiation with an operating voltage of 45 kV and current of 40 mA, at room temperature, to study the crystal structure of the electrospun fibers. Part of the sample was ground before the XRD measurement so that a powder pattern could be obtained for phase identification. Scanning electron microscopy (SEM) measurements were performed on a Jeol SEM-6500 to study the morphologies of the fibers. Energy dispersive X-ray spectroscopic (EDXS) analysis and scanning transmission electron microscopy (STEM) mapping of the sample were carried out using the same equipment for SEM imaging to study the elemental composition of the fibers. Magnetic measurements were made using a magnetic property measurement system (MPMS) from Quantum Design. The sample was sealed in a gelatine capsule and placed inside a straw and then loaded into the MPMS.

## Results and Analysis

**Figure 2:**
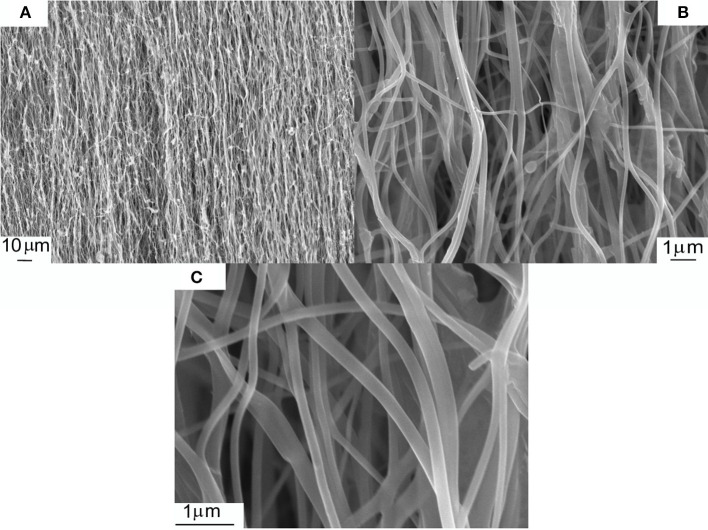
SEM micrographs of PVP/ Fe nitrate/Ni acetate electrospun nanofibers. **(A–C)** show different magnifications of the same sample.

It can be seen in the SEM micrographs in Figure 2A that electrospinning has resulted in orientated PVP/Fe nitrate/Ni acetate fibers. A higher magnification micrograph in Figure 2B shows that there are some nanofibers that were not in the general orientated direction and that there is also some fusing of nanofibers. Figure 2C shows that the nanofibre diameters range from ~200 to ~250 nm.

**Figure 3:**
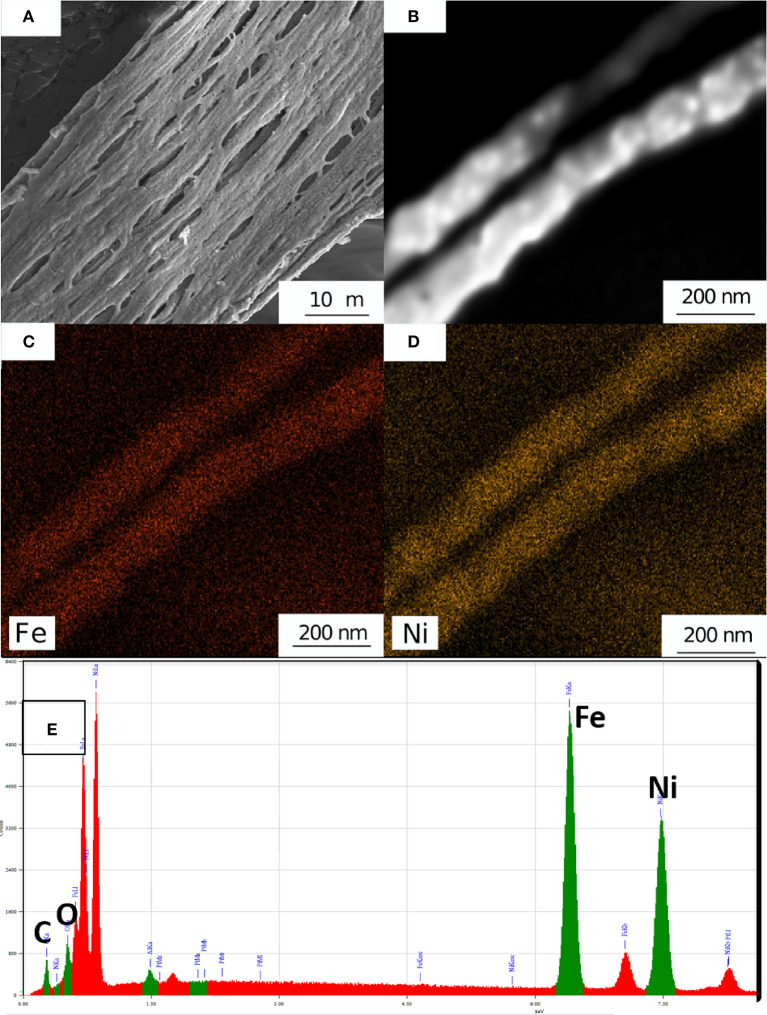
**(A)** SEM micrograph after high temperature processing in an Argon/H_2_ atmosphere leading to a Ni_1-*x*_Fe_*x*_ mat containing submicron fibers. **(B)** A SEM micrograph of two Ni_1-*x*_Fe_*x*_ fibers and STEM elemental maps of **(C)** Fe and **(D)** Ni. EDX spectra of Ni_1-*x*_Fe_*x*_ fibers.

The sample morphology of the electrospun fibers dramatically changed after processing at 600°C in 95% Ar/5% H_2_ as can be seen in Figure 3A. It is apparent that the fibers seen before processing have coalesced to form larger oriented submicron fibers with diameters ranging from ~600 to ~900 nm. These fibers have fused to create a mat with significant voids. There are a few small branching nanofibers with diameters in the ~300 nm range. A small area (Figure 3B) was selected for scanning transmission electron microscopy (STEM) mapping and the STEM maps are shown in Figure 3C for Fe and Figure 3D for Ni are shown in Figure 3B. It can be seen that Fe and Ni are uniformly distributed within the fiber. Energy dispersive X-ray spectroscopy (EDXS) showed that the fibers were Ni_1-*x*_Fe_*x*_ with *x* = 0.53 (± 0.03), which is the value expected from the initial precursor ratio (Figure 3E). The carbon peak was small and shows that there was removal of significant fraction of the polymer. The low carbon signal was likely from the SEM sample handling and processing (for example, the adhesive on the tape holding the sample on SEM stub).

**Figure 4:**
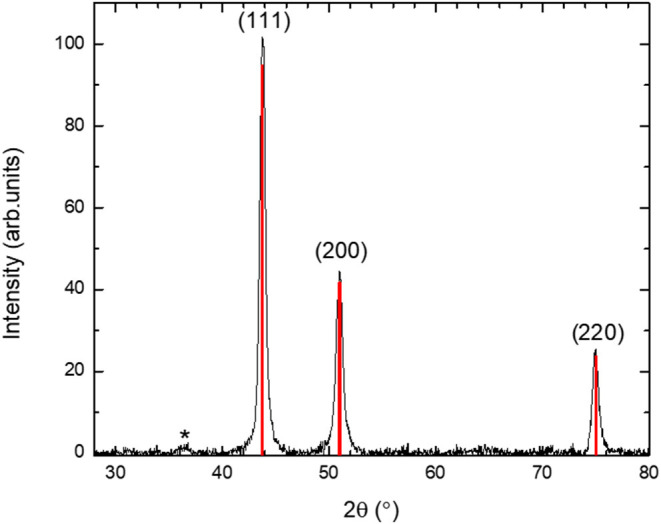
XRD data for the Ni_1-*x*_Fe_*x*_ mat containing submicron fibers after grinding. Also shown are the Miller indices for FCC Ni_1-*x*_Fe_*x*_. A weak impurity phase is indicated by an asterisk and it is in the region where the main FCC FeO and NiO XRD peaks are expected.

**Figure 5:**
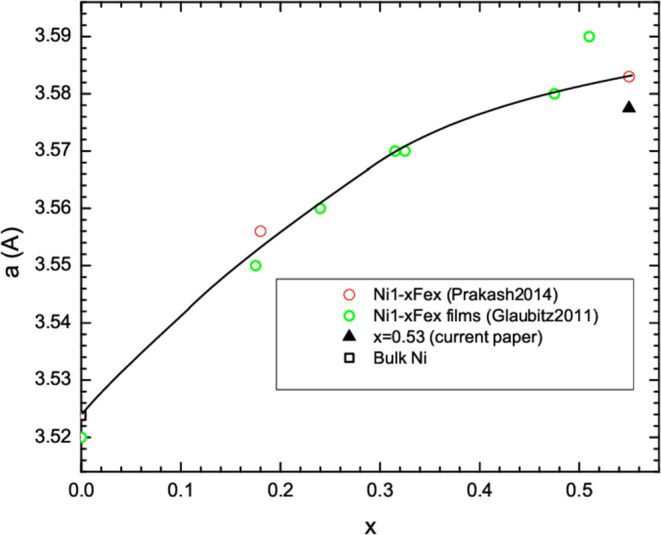
Fitting of XRD data for lattice parameter comparison with previous literature.

The results from XRD measurements after the Ni_0.47_Fe_0.53_ nanofibers synthesis are shown in Figure 4. The main phase can be fitted to FCC Ni_1-*x*_Fe_*x*_, which was expected for *x* < 0.6 (Li et al., 1997). Fitting of the XRD data shows that the lattice parameter was *a* = 3.5775 Å. By comparison with data in the literature (Glaubitz et al., 2011; Prakash et al., 2014), we found that this lattice parameter is in the range expected for *x* = 0.53 (Figure 5). Since XRD is an averaging probe over the whole sample, this, when combined with the EDXS and STEM map data, shows that the *x* content did not significantly vary across the sample. A very small and broad peak was found at 2θ of ~36.5°. This may be due to a small fraction of Fe_1-*z*_Ni_*z*_O because FCC NiO and FeO have (111) peaks in this region.

Broadening of the XRD peaks in Figure 4 was observed, which arises in materials with small nanocrystals and/or strain. For this reason, the XRD peaks were fitted to the pseudo Voight functions to obtain the linewidth. A Williamson-Hall analysis (Birkholz et al., 2006) showed that there is negligible strain and the Ni_0.47_Fe_0.53_ fibers were composed of nanocrystals with average diameters of ~14 nm.

**Figure 6:**
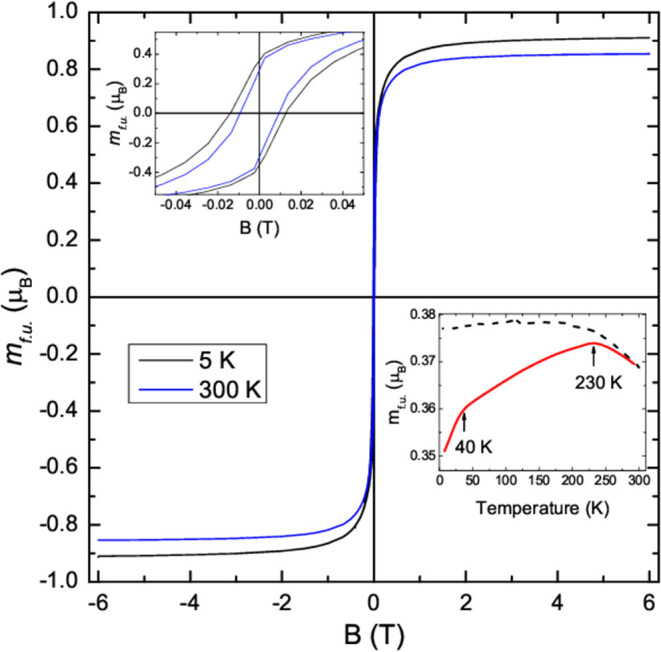
Plot of the moment per formula unit, *m*_*f*.*u*__._, against temperature at 5 K (black curve) and 300 K (blue curve). An expanded view is plotted in the upper right inset. Lower left plot: plot of the ZFC and FC m_f.u._ against temperature for an applied magnetic field of 10 mT.

Magnetic measurements were performed on the as-made Ni_0.47_Fe_0.53_ fiber mats. The moment per formula unit, *m*_*f*.*u*._, was plotted as a function of applied magnetic field, *B*, in Figure 6 at 5 and 300 K. *m*_*f*.*u*__._ was plotted in Bohr magneton units, μ_B_. *m*_*f*.*u*_ was calculated as the moment/(no. of moles x Avogadro's no.). The data clearly shows the presence of ferromagnetic order that is expected for Ni_1-*x*_Fe_*x*_. The high field magnetic moment, *m*_*f*.*u*.,*sat*_, was large and it was 0.91 μ_B_ at 5 K. This was less than typical bulk samples, where *m*_*f*.*u*.,*sat*_ = 1.78 μ_B_ for Ni_1-*x*_Fe_*x*_ with *x* = 0.53 (Li et al., 1997). It is comparable to that reported for ~2.7 nm diameter Ni_1-*x*_Fe_*x*_ nanoparticles with *x* = 0.47 and made by dual ion beam implantation where *m*_*f*.*u*.,*sat*_= 1.0 μ_B_ (Williams et al., 2019). Nanoscale magnetic materials typically have lower *m*_*f*.*u*.,*sat*_ than the bulk (Vitta et al., 2008; Demortière et al., 2011; Upadhyay et al., 2016; Williams et al., 2018).

The upper left inset to Figure 6 shows that the coercive field was enhanced when compared with the bulk compound where the coercive field and the saturation field are small. The enhanced coercivity is likely to be due a high density of domain wall pinning sites at the nanograin boundaries. A larger coercivity was observed at low temperatures because domain wall motion is thermally activated as well as occurring via the applied magnetic field. The zero-field-cooled (ZFC) and field-cooled (FC) *m*_*f*.*u*__._ is plotted in the lower right inset to Figure 6. The ZFC data were taken after cooling in zero applied field to low temperatures. A magnetic field of 10 mT was then applied and magnetic measurements were taken while warming up to 300 K followed by field-cooling down to low temperatures. Magnetic hysteresis is still evident at the highest temperature and indicates that the nanocrystals are too large for superparamagnetism to occur.

Two features can be seen in the ZFC *m*_*f*.*u*__._ data. The first is a peak at ~230 K and the second is a faster decrease below ~40 K. The ~230 K peak was likely to arise from a small antiferromagnetic phase fraction. It is too small to be purely from NiO, which has a Neel temperature of 525 K. It is also higher than that seen in bulk FeO where the Neel temperature is 198 K. Thus, it may have been arisen from a Fe_1-*z*_Ni_*z*_O phase that is seen in the XRD data and that would be expected to have a Neel temperature between 198 and 525 K. The more rapid decrease below 40 K may be related to a small spin-disorder region that is discussed below.

**Figure 7:**
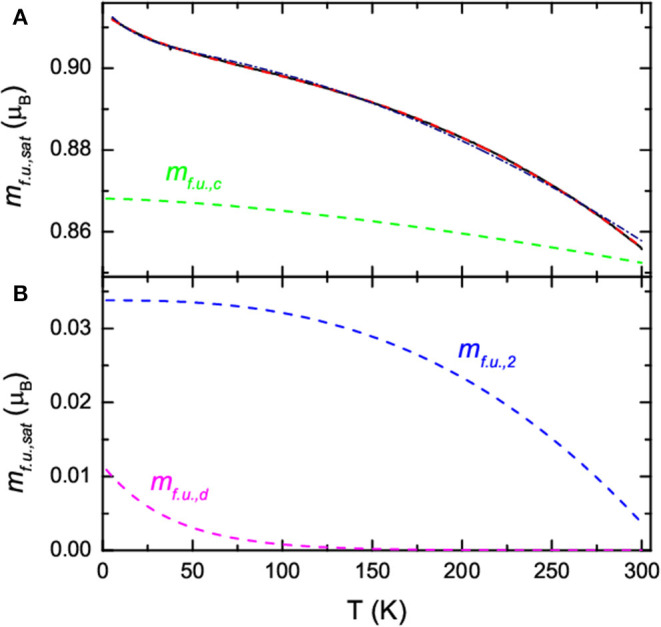
**(A)** Plot of the saturation moment per formula unit, *m*_*f*.*u,sat*__._, against temperature at 5 K; (solid black curve) a fit to the data using Equation (2) (dot dashed navy curve) and Equation (3) (dashed red curve). The ordered core contribution to the fit, *m*_*f*.*u*.,*c*_, using Equation (3) is also plotted (dashed green curve). **(B)** Spin-disordered, *m*_*f*.*u*.,*d*_, (dashed magenta curve) and the 2nd contribution, *m*_*f*.*u*.,__2_, (blue dashed curve) to the fit to the data in **(A)** using Equation (3).

The saturation magnetic moment per formula unit, *m*_*f*.*u*.,*sat*_, obtained from the moment per formula unit at 6 T, is plotted in Figure 7A as a function of temperature. The temperature dependence of *m*_*f*.*u*.,*sat*_ for bulk Ni_1-*x*_Fe_*x*_ is known to follow the Bloch function (Bloch, 1930; Ashcroft and Mermin, 1976) that can be written as,

(1) $$\begin{array}{l} m_{f.u.,sat}\left(T\right)=m_{f.u.,sat}\left(0\right)\left[1-\beta \times T^{n}\right] \end{array}$$

where *n* is the exponent, *T* is the temperature, *m*_*f*.*u*.,*sat*_(0) is *m*_*f*.*u*.,*sat*_ at 0 K, and β is the Bloch constant. *n* = 3/2 is expected for Ni_1-*x*_Fe_*x*_ and for simple ferromagnetic materials (Ashcroft and Mermin, 1976). β is proportional to 1/*D*^3/2^ where *D* is the spin stiffness that in turn is proportional to the exchange energy (Srivastava and Aiyar, 1987; Demortière et al., 2011).

It is clear in Figure 7A that *m*_*f*.*u*.,*sat*_ does not follow Equation (1) due to the upturn at lower temperatures. This type of behavior has been reported before in ferromagnetic nanoparticles (Vázquez-Vázquez et al., 2011; Larumbe et al., 2012; Williams et al., 2019). It has been shown that *m*_*f*.*u*.,*sat*_ can be fitted to (Vázquez-Vázquez et al., 2011; Larumbe et al., 2012),

(2) $$\begin{array}{l} m_{f.u.,sat}\left(T\right)=m_{f.u.,c}\left(0\right)\times \left[1-\beta \times T^{n}\right]+m_{f.u.,d}\left(0\right)\\ \;\times \text{exp}\left(-T/T_{f}\right) \end{array}$$

where the first term is the Bloch function and the second term is a phenomenological term to account for spin-disorder in the shell region. *m*_*f*.*u*.,*c*_(0) is the saturation moment per formula unit in the core at 0 K, *m*_*f*.*u*.,*d*_(0) is the spin-disordered saturation moment per formula unit at 0 K, and *T*_*f*_ is the characteristic spin-freezing temperature.

We showed in Figure 7A that Equation (2) provides and an approximate fit to the data (navy dot dashed curve) with *m*_*f*.*u*.,*c*_(0) = 0.905 μ_B_, β = 2.0 × 10^−6^ K^−1.78^, *n* = 1.78, *m*_*f*.*u*.,*d*_(0) = 0.0099 μ_B_, and *T*_*f*_ = 17 K. However, the value of *n* is larger than that seen in the bulk or Ni_1-*x*_Fe_*x*_ nanoparticles with diameters >35 nm (Vitta et al., 2008). It is possible that the larger *n* and approximate fit to the data is due to a small contribution from another magnetically ordered phase. This could be Fe_1-*z*_Ni_*z*_O that can have a measurable magnetic moment due to the moments not being colinear. In this case, an extra term is added to Equation (2) leading to

(3) $$\begin{array}{l} m_{f.u.,sat}\left(T\right)=m_{f.u.,c}\left(0\right)\times \left[1-\beta \times T^{n}\right]+m_{f.u.,d}\left(0\right)\\ \;\times \text{exp}\left(\frac{-T}{T_{f}}\right)+m_{f.u.,2}\left(0\right)\times \left[1-\beta_{2}\times T^{n2}\right] \end{array}$$

where *m*_*f*.*u*.,__2_(0) is the saturation moment per formula unit from the second smaller phase fraction at 0 K, β_2_ is the temperature prefactor, and *n*_2_ is the temperature exponent.

We showed in Figure 7 that Equation (3) provides an excellent fit to the data (red dashed curve) with *m*_*f*.*u*.,*c*_(0) = 0.868 μ_B_, β = 3.5 × 10^−6^ K^−1.5^, *n* = 1.5, *m*_*f*.*u*.,*d*_(0) = 0.011 μ_B_, *T*_*f*_ = 38 K, *m*_*f*.*u*.,__2_(0) = 0.034 μ_B_, β_2_ = 3.0 × 10^−6^ K^−2.6^, and *n*_2_ = 2.6. In this case *n* is the same as that found in the bulk and in Ni_1-*x*_Fe_*x*_ nanoparticles (Vitta et al., 2008; Williams et al., 2019), and β is in the range found for bulk Ni_1-*x*_Fe_*x*_ (Zhang et al., 1998; Vitta et al., 2008). The value of *T*_*f*_ is where a faster decrease in the ZFC *m*_*f*.*u*._ was observed. Figures 7A,B also show the contribution to the total fit from the three terms in Equation (3). The dominant contribution is from the ferromagnetic Ni_1-*x*_Fe_*x*_ core (green dashed curve in Figure 7A). The spin-disordered contribution is small.

The low value of *m*_*f*.*u*.,*d*_(0) suggests that it is unlikely that the spin-disordered contribution is from the shell regions between the Ni_1-*x*_Fe_*x*_ nanocrystals. A simple estimate of the shell thickness can be obtained using a model developed for nanoparticles were the average spin-disordered shell thickness, *t*_*shell*_ can be estimated from *t*_*shell*_ = (1–γ^1/3^) × *r* where γ = *m*_*f*.*u*.,*c*_(0)/[ *m*_*f*.*u*.,*c*_(0) + *m*_*f*.*u*.,*d*_(0)] and *r* is the average radius of the nanocrystals (Prakash et al., 2016). We find that *t*_*shell*_ is only 0.03 nm for an average *r* of 7 nm, which is unrealistically small. It is possible that the spin-disordered region surrounds the Ni_1-*x*_Fe_*x*_ fibers. In this case the simple model gives,

(4) $$\begin{array}{l} t_{shell}=\left(1-\gamma^{1/2}\right)\times r \end{array}$$

where *t*_*shell*_ is now thickness of the spin-disordered region surrounding the fibers and r is the radius of the fibers. Using an average fiber diameter of 750 nm, this gives *t*_*shell*_ = 2.3 nm, which is plausible.

The temperature dependence of the additional contribution to the fit to the data in Figure 7A was plotted in Figure 7B (blue dashed curve). This contribution is small and close to zero at 300 K. This suggests a magnetic phase with a magnetic ordering temperature at or slightly below 300 K. It may be that it is due to Fe_1-*z*_Ni_*z*_O surrounding the fibers with a range of magnetic ordering temperatures up to ~300 K. In this case an estimate of the average thickness of the oxidized region surrounding the fibers can be obtained from Equation (4). Using an average fiber diameter of 750 nm, we find that *t*_*shell*_ ~ 7 nm.

## Conclusions

In conclusion, ferromagnetic Ni_0.47_Fe_0.53_ mats containing orientated Ni_0.47_Fe_0.53_ nanofibers were fabricated by electrospinning solutions of ferric nitrate and nickel acetate metal precursors in polyvinylpyrrolidone (PVP) polymer solutions followed by thermal processing. The final synthesis step at 600°C in 95% Ar:5% H_2_ led to the complete removal of the polymer and only Ni_0.47_Fe_0.53_ fibers and a small fraction of Fe_1-*z*_Ni_*z*_O remained. There were a range of Ni_0.47_Fe_0.53_ nanofibre diameters that varied from ~600 to ~900 nm where they had fused to form the Ni_0.47_Fe_0.53_ mats. The fibers were nanostructured and contained nanocrystals with average diameters of ~14 nm as estimated from the XRD data. Magnetic measurements clearly showed the presence of ferromagnetic order. The saturation magnetic moment per formula was less than that found in the bulk but it was similar to that reported in other studies of nanostructured Ni_1-*x*_Fe_*x*_ with similar *x* values. Modeling of the temperature dependence of the saturation moment showed that there was some spin-disorder, probably from a thin layer in the surface region of the fibers, with a characteristic spin-freezing temperature of 38 K. There was also an additional minor magnetically ordered phase with a magnetic ordering temperature below room temperature that may be from a thin antiferromagnetic Fe_1-*z*_Ni_*z*_O layer surrounding the nanofibers. The coercivity was enhanced when compared with the bulk, which is probably due to the small nanocrystals that lead to a high density of domain wall pinning centers.

## Data Availability Statement

The datasets generated for this study are available on request to the corresponding author.

## Author Contributions

FH undertook initial experiments to electrospin magnetic nanofibers. VB undertook the electrospinning, SEM, and XRD measurements. GW did the XRD and magnetic analysis and interpretation. SC did the magnetic measurements and assisted in the magnetic interpretation. TN conceived and helped to oversee the research. All authors were involved in the data interpretation.

### Conflict of Interest

The authors declare that the research was conducted in the absence of any commercial or financial relationships that could be construed as a potential conflict of interest.

## References

[B1] Ashcroft N. W. Mermin N. D. (1976). Solid State Physics. New York, NY: Holt, Rinehart and Winston.

[B2] Balaji G. Narayanan R. A. Weber A. Mohammad F. Kumar C. S. S. R. (2012). Giant magnetostriction in magnetite nanoparticles. Mater. Sci. Eng. B 177, 14–18. 10.1016/j.mseb.2011.09.023

[B3] Barakat N. A. M. Kim B. Kim H. Y. (2009). Production of smooth and pure nickel metal nanofibers by the electrospinning technique: nanofibers possess splendid magnetic properties. J. Phys. Chem. C 113, 531–536. 10.1021/jp805692r

[B4] Batlle X. Labarta A. (2002). Finite-size effects in fine particles: magnetic and transport properties. J. Phys. D Appl. Phys. 35, R15–R42. 10.1088/0022-3727/35/6/201

[B5] Birkholz M. Fewster P. F. Genzel C. (2006). Thin Film Analysis by X-Ray Scattering. Weinheim: Wiley-VCH.

[B6] Bloch F. (1930). Zur theorie des ferromagnetismus. Zeitsch. Phys. 61, 206–219. 10.1007/BF01339661

[B7] Bozorth R. M. Walker J. G. (1953). Magnetic crystal anisotropy and magnetostriction of iron-nickel alloys. Phys. Rev. 89, 624–628. 10.1103/PhysRev.89.624

[B8] Chen L. X. Huang X. G. Zhu J. H. Li G. C. Lan S. (2011). Fiber magnetic-field sensor based on nanoparticle magnetic fluid and Fresnel reflection. Opt. Lett. 36:2761. 10.1364/OL.36.002761 21808304

[B9] Chinnasamy C. Malallah Y. Jasinski M. M. Daryoush A. S. (2015). Synthesis of high magnetic moment soft magnetic nanocomposite powders for RF filters and antennas. Appl. Surf. Sci. 334, 58–61. 10.1016/j.apsusc.2014.08.025

[B10] Chong S. V. Williams G. V. M. (2019). Magnetoelectric effect in magnetostrictive-piezoelectric composites containing magnetite nanoparticles. Sens. Actuators A Phys. 288, 101–106. 10.1016/j.sna.2019.02.003

[B11] Cisternas E. Faúndez J. Vogel E. E. (2017). Stabilization mechanisms for information stored in magnetic nanowire arrays. J. Magnetism Magn. Mater. 426, 588–593. 10.1016/j.jmmm.2016.11.022

[B12] Cullity B. D. Graham C. D. (2009). Introduction to Magnetic Materials, 2nd Edn. Hoboken, NJ: IEEE/Wiley.

[B13] Daughton J. Brown J. Chen E. Beech R. Pohm A. Kude W. (1994). Magnetic field sensors using GMR multilayer. IEEE Transac. Magn. 30, 4608–4610. 10.1109/20.334164

[B14] Demortière A. Panissod P. Pichon B. P. Pourroy G. Guillon D. Donnio B.. (2011). Size-dependent properties of magnetic iron oxidenanocrystals. Nanoscale 3, 225–232. 10.1039/c0nr00521e 21060937

[B15] Dong X. Qi M. Tong Y. Ye F. (2014). Solvothermal synthesis of single-crystalline hexagonal cobalt nanofibers with high coercivity. Mater. Lett. 128, 39–41. 10.1016/j.matlet.2014.04.133

[B16] Frolov K. V. Chuev M. A. Lyubutin I. S. Zagorskii D. L. Bedin S. A. Perunov I. V. (2019). Structural and magnetic properties of Ni-Fe nanowires in the pores of polymer track membranes. J. Magn. Magn. Mater. 489:165415 10.1016/j.jmmm.2019.165415

[B17] Glaubitz B. Buschhorn S. Brüssing F. Abrudan R. Zabel H. (2011). Development of magnetic moments in Fe_1−x_ Ni_x_ -alloys. J. Phys. Condens. Matter 23:254210 10.1088/0953-8984/23/25/254210

[B18] Goya G. F. Berquó T. S. Fonseca F. C. Morales M. P. (2003). Static and dynamic magnetic properties of spherical magnetite nanoparticles. J. Appl. Phys. 94, 3520–3528. 10.1063/1.1599959

[B19] Graeser M. Bognitzki M. Massa W. Pietzonka C. Greiner A. Wendorff J. H. (2007). Magnetically anisotropic cobalt and iron nanofibers via electrospinning. Adv. Mater. 19, 4244–4247. 10.1002/adma.200700849

[B20] Guo-Min Y. Xing X. Daigle A. Liu M. Obi O. Stoute S. (2009). Tunable miniaturized patch antennas with self-biased multilayer magnetic films. IEEE Trans. Antennas Propagation 57, 2190–2193. 10.1109/TAP.2009.2021972

[B21] Hendriksen P. V. Linderoth S. Lindgård P.-A. (1993). Finite-size modifications of the magnetic properties of clusters. Phys. Rev. B 48, 7259–7273. 10.1103/PhysRevB.48.7259 10006894

[B22] Inoue J. Maekawa S. (1996). Theory of tunneling magnetoresistance in granular magnetic films. Phys. Rev. B 53, R11927–R11929. 10.1103/PhysRevB.53.R11927 9982889

[B23] Katti R. R. (2002). Current-in-plane pseudo-spin-valve device performance for giant magnetoresistive random access memory applications (invited). J. Appl. Phys. 91:7245 10.1063/1.1456037

[B24] Kennedy J. Leveneur J. Turner J. Futter J. Williams G. V. M. (2014). “Applications of nanoparticle-based fluxgate magnetometers for positioning and location,” in 2014 IEEE Sensors Applications Symposium (SAS) (Queenstown: IEEE), 228–232.

[B25] Kim E. H. Ahn Y. Lee H. S. (2007). Biomedical applications of superparamagnetic iron oxide nanoparticles encapsulated within chitosan. J. Alloys Comp. 434–435, 633–636. 10.1016/j.jallcom.2006.08.311

[B26] Larumbe S. Gómez-Polo C. Pérez-Landazábal J. I. Pastor J. M. (2012). Effect of a SiO _2_ coating on the magnetic properties of Fe_3_O_4_ nanoparticles. J. Phys. Condens. Matter 24:266007 10.1088/0953-8984/24/26/266007 22700683

[B27] Li D. Herricks T. Xia Y. (2003). Magnetic nanofibers of nickel ferrite prepared by electrospinning. Appl. Phys. Lett. 83, 4586–4588. 10.1063/1.1630844

[B28] Li X. G. Chiba A. Takahashi S. (1997). Preparation and magnetic properties of ultrafine particles of Fe-Ni alloys. J. Magn. Magn. Mater. 170, 339–345. 10.1016/S0304-8853(97)00039-5

[B29] Meneses F. Urreta S. E. Escrig J. Bercoff P. G. (2018). Temperature dependence of the effective anisotropy in Ni nanowire arrays. Curr. Appl. Phys. 18, 1240–1247. 10.1016/j.cap.2018.06.014

[B30] Moser A. Takano K. Margulies D. T. Albrecht M. Sonobe Y. Ikeda Y. (2002). Magnetic recording: advancing into the future. J. Phys. D Appl. Phys. 35, R157–R167. 10.1088/0022-3727/35/19/201

[B31] Nogués J. Sort J. Langlais V. Skumryev V. Suriñach S. Muñoz J. S. (2005). Exchange bias in nanostructures. Phys. Rep. 422, 65–117. 10.1016/j.physrep.2005.08.004

[B32] Pankhurst Q. A. Connolly J. Jones S. K. Dobson J. (2003). Applications of magnetic nanoparticles in biomedicine. J. Phys. D Appli. Phys. 36, R167–R181. 10.1088/0022-3727/36/13/201

[B33] Parkin S. S. P. Kaiser C. Panchula A. Rice P. M. Hughes B. Samant M.. (2004). Giant tunnelling magnetoresistance at room temperature with MgO (100) tunnel barriers. Nat. Mater. 3, 862–867. 10.1038/nmat1256 15516928

[B34] Petrov R. V. Tatarenko A. S. Pandey S. Srinivasan G. Mantese J. V. Azadegan R. (2008). Miniature antenna based on magnetoelectric composites. Electron. Lett. 44:506 10.1049/el:20080325

[B35] Prakash T. Williams G. V. M. Kennedy J. Murmu P. P. Leveneur J. Chong S. V. (2014). Synthesis and structural, magnetic and magnetotransport properties of permalloy powders containing nanoparticles prepared by arc discharge. J. Alloys Comp. 608, 153–157. 10.1016/j.jallcom.2014.04.111

[B36] Prakash T. Williams G. V. M. Kennedy J. Rubanov S. (2016). Formation of magnetic nanoparticles by low energy dual implantation of Ni and Fe into SiO_2_. J. Alloys Comp. 667, 255–261. 10.1016/j.jallcom.2016.01.172

[B37] Salem M. S. Sergelius P. Zierold R. Montero Moreno J. M. Görlitz D. Nielsch K. (2012). Magnetic characterization of nickel-rich NiFe nanowires grown by pulsed electrodeposition. J. Mater. Chem. 22:8549 10.1039/c2jm16339j

[B38] Srivastava C. M. Aiyar R. (1987). Spin wave stiffness constants in some ferrimagnetics. J. Phys. C Solid State Phys. 20, 1119–1128. 10.1088/0022-3719/20/8/013

[B39] Upadhyay S. Parekh K. Pandey B. (2016). Influence of crystallite size on the magnetic properties of Fe_3_O_4_ nanoparticles. J. Alloys Comp. 678, 478–485. 10.1016/j.jallcom.2016.03.279

[B40] Vázquez-Vázquez C. López-Quintela M. A. Buján-Núñez M. C. Rivas J. (2011). Finite size and surface effects on the magnetic properties of cobalt ferrite nanoparticles. J. Nanoparticle Res. 13, 1663–1676. 10.1007/s11051-010-9920-7

[B41] Vitta S. Khuntia A. Ravikumar G. Bahadur D. (2008). Electrical and magnetic properties of nanocrystalline Fe100–xNix alloys. J. Magn. Magn. Mater. 320, 182–189. 10.1016/j.jmmm.2007.05.021

[B42] Williams G. V. M. Kennedy J. Murmu P. P. Rubanov S. Chong S. V. (2019). The effect of different Fe concentrations on the structural and magnetic properties of near surface superparamagnetic Ni1–Fe nanoparticles in SiO_2_ made by dual low energy ion implantation. J. Magn. Magn. Mater. 473, 125–130. 10.1016/j.jmmm.2018.10.072

[B43] Williams G. V. M. Prakash T. Kennedy J. Chong S. V. Rubanov S. (2018). Spin-dependent tunnelling in magnetite nanoparticles. J. Magn. Magn. Mater. 460, 229–233. 10.1016/j.jmmm.2018.04.017

[B44] Wu G. Nan T. Zhang R. Zhang N. Li S. Sun N. X. (2013). Inequivalence of direct and converse magnetoelectric coupling at electromechanical resonance. Appl. Phys. Lett. 103:182905 10.1063/1.4827875

[B45] Wu H. Zhang R. Liu X. Lin D. Pan W. (2007). Electrospinning of Fe, Co, and Ni nanofibers: synthesis, assembly, and magnetic properties. Chem. Mater. 19, 3506–3511. 10.1021/cm070280i

[B46] Zhang D. Klabunde K. J. Sorensen C. M. Hadjipanayis G. C. (1998). Magnetization temperature dependence in iron nanoparticles. Phys. Rev. B 58, 14167–14170. 10.1103/PhysRevB.58.14167

[B47] Zhang X. Zhang H. Wu T. Li Z. Zhang Z. Sun H. (2013). Comparative study in fabrication and magnetic properties of FeNi alloy nanowires and nanotubes. J. Magn. Magn. Mater. 331, 162–167. 10.1016/j.jmmm.2012.11.033

[B48] Žutić I. Fabian J. Das Sarma S. (2004). Spintronics: fundamentals and applications. Rev. Mod. Phys. 76, 323–410. 10.1103/RevModPhys.76.323

