# Corrigendum: Electrospun, Oriented, Ferromagnetic Ni_*1-x*_Fe_*x*_ Nanofibers

**DOI:** 10.3389/fchem.2020.00809

**Published:** 2020-09-03

**Authors:** Vaibhav S. Bhugra, Fraser R. Hughson, Grant V. M. Williams, Shen V. Chong, Thomas Nann

**Affiliations:** ^1^School of Chemical and Physical Sciences, Victoria University of Wellington, Wellington, New Zealand; ^2^Robinson Research Institute, Victoria University of Wellington, Lower Hutt, New Zealand; ^3^School of Mathematical and Physical Sciences, The University of Newcastle, Newcastle, NSW, Australia

**Keywords:** magnetic properties, electrospinning, nanofibers, ferromagnetic, polyvinylpyrrolidone (PVP), nanomaterials

Fraser R. Hughson was not included as an author in the published article. The corrected Author Contributions Statement appears below.

FH undertook initial experiments to electrospin magnetic nanofibers. VB undertook the electrospinning, SEM, and XRD measurements. GW did the XRD and magnetic analysis and interpretation. SC did the magnetic measurements and assisted in the magnetic interpretation. TN conceived and helped to oversee the research. All authors were involved in the data interpretation.

The authors apologize for this error and state that this does not change the scientific conclusions of the article in any way. The original article has been updated.

